# Process evaluation of Tuberculosis infection control and prevention practice at public health facilities in Tegede district, Northwest Ethiopia: Facility-based cross-sectional design

**DOI:** 10.1371/journal.pone.0314514

**Published:** 2025-02-14

**Authors:** Endalkachew Mesfin Gebeyehu, Ashagere Mebratu, Asmamaw Atnafu, Asebe Hagos

**Affiliations:** 1 Department of Health System Management, School of Public Health, College of Medicine and Health Sciences, Wollo University, Dessie, Ethiopia; 2 Department of Health System and Policy, College of Medicine and Health Sciences, University of Gondar, Gondar, Ethiopia; The University of Georgia, UNITED STATES OF AMERICA

## Abstract

**Background:**

Tuberculosis Infection Prevention and Control practice (TB-IPC) is crucial to prevent nosocomial transmission of Tuberculosis (TB), especially in high-burden settings like Ethiopia. However, studies show that TB infection control practices among healthcare workers in Ethiopia are suboptimal. The study aims to evaluate TB-IPC practices at public health facilities in Tegede district, Northwest Ethiopia.

**Methods:**

A facility-based cross-sectional design with mixed methods and a formative approach was used to evaluate the TB-IPC from May 1 to June 30, 2023. The practice was assessed using availability, compliance, and acceptability dimensions with 31 indicators. The quantitative data was collected from 525 clients using structured questionnaires. The data were coded, entered into EpiData version 4.6, and exported to SPSS version 25 for analysis. Binary and multivariable logistic regression analyses were used to identify the predictor variables associated with client satisfaction. Furthermore, non-participatory direct observations, document reviews, and resource inventories were conducted. In the qualitative study, key informants were interviewed, and qualitative data were transcribed, translated, coded, and analyzed manually in themes.

**Result:**

The overall implementation of TB-IPC practice was 54.4%. The availability dimension was 50.4%, compliance was 67.2%, and acceptability was 43.4%. A total of 525 clients participated, with a response rate of 96.2%. In the multivariable logistic regression analysis, occupation was the predicator variable with a p-value of <0.05. In total, 134 (25.5%) clients, with a 95% CI [22.0%, 29.4%], were satisfied with TB-IPC practices at public health centers in Tegede district.

**Conclusion:**

The overall implementation of TB-IPC practices at public health facilities in Tegede district is currently poor and requires urgent improvement. Based on stakeholders’ judgment criteria, the dimensions of availability, compliance, and acceptability were rated as poor, fair, and poor, respectively. These findings provide a baseline understanding of how TB-IPC practices are implemented in Tegede district.

## Background

Tuberculosis is one of the top ten causes of death worldwide and the leading cause of death from a single source of infection [1]. According to the 2023 WHO Global TB Report, Ethiopia reported approximately 10 million TB infections globally, with an estimated 1.6 million deaths due to TB in 2021. The incidence of TB in Ethiopia was reported at 172 cases per 100,000 population, and the TB/HIV co-infection rate was approximately 12% [2].

The World Health Organization’s (WHO) End TB Strategy, approved by the World Health Assembly in 2014 and updated on December 16, 2022, calls for a 90% reduction in TB deaths and an 80% decrease in the TB incidence rate by 2030 using 2015 as a baseline. The strategy emphasizes the need for prevention across all approaches, including infection prevention and control (IPC) in health care services and other settings where the risk of *Mycobacterium tuberculosis* transmission is high [3].

Tuberculosis remains a major global public health threat, and the End TB Strategy and United Nations targets for TB are all progress towards targets are behind schedule. The COVID-19 pandemic aggravated an already suboptimal global TB response, so achieving TB targets will require optimized use of existing tools and new tools [4]. In Ethiopia, TB infection prevention and control are growing in importance because of the association of TB with HIV and the emergence of DR-TB. An increasing number of patients, lack of infrastructure, and insufficient healthcare staffing has led to overcrowding, delays in diagnosis, and inadequate treatment, resulting in increased TB transmission [5].

The major drivers of TB remain undernutrition, poverty, diabetes, tobacco smoking, and household air pollution, and these need to be addressed to achieve the WHO 2035 TB care and prevention targets [6]. Community-based and community-led responses that take diagnosis, care, and support to the doors of those affected have much potential. For example, people with respiratory symptoms and illnesses should be able to receive community-based contact tracing, directly observed therapy, isolation, and quarantine support from trusted friends, family, and neighbors [7].

Ethiopia is one of the most TB-burden countries, and TB remains a challenging problem even though the Ethiopian government has been working over the past decades to enhance TB-IPC activities. Even though global and national guidelines have been published, and healthcare workers (HCWs) have received training, TB-IPC practices have been assessed in various regions of Ethiopia, including the Tegede region. Throughout Ethiopia, the rates of implementing TB-IPC measures have varied widely. Studies have shown that implementation rates range from 34% in health centers in Southern Ethiopia [8] to 74% in hospitals in Addis Ababa [9]. These variations highlight the challenges in standardizing TB-IPC practices across different healthcare settings.

Ethiopia is among the 30 high-TB burden countries identified by the WHO. According to the WHO, Ethiopia had an estimated 151,000 new TB cases in 2021, with a significant number of these cases being drug-resistant. The high prevalence underscores the need for robust TB infection control practices to prevent transmission, especially in high-risk areas such as healthcare facilities and densely populated communities [10]. Patients and providers are therefore at risk of exposure to TB, especially at high-case-load health facilities where TB patients and suspected cases stay longer for inpatient or outpatient follow-up care. The risk to others is magnified if the patients do not receive the proper treatment and IPC guidelines are not followed. Tuberculosis is vastly contagious in resource-limited healthcare facilities, and Health Care Workers (HCWs) are at increased risk of acquiring TB in such settings [11].

Tuberculosis infection control is a combination of measures designed to minimize the risk of TB transmission within populations. Healthcare workers are not sufficiently protected from tuberculosis infection in healthcare facilities where infection control protocols are not followed completely [3].

There are health system influences on TB-IPC implementation referred to as availability, knowledge, and educational development of staff, timeliness of service delivery, availability of equipment, such as respirators and masks, space for patient separation, funding, and TB-IPC information, education, and communication materials and tools (8). One of the biggest problems with local community health continues to be the airborne transmission of TB. In the district health institutions, tuberculosis infection prevention and control procedures have long been implemented ineffectively.

The evaluation of TB-IPC practice at public health facilities in Tegede district, Northwest Ethiopia, was necessary for several reasons: TB is highly prevalent in sub-Saharan Africa, including Ethiopia, making the risk of infection transmission high in these countries. Effective TB-IPC practices are crucial to prevent nosocomial transmission of TB among patients and healthcare workers [12, 13]. Healthcare workers are at risk of exposure to TB, especially at high-caseload facilities. Therefore, it is essential to assess and improve TB-IPC practices to prevent transmission and ensure patient safety [14]. The aim was not solely driven by stakeholders’ demands. However, the evaluation of TB-IPC practices was initiated at their request to identify gaps and areas for improvement. The main stakeholders for this program are described in Table 1.

**Table 1:** Stakeholder analysis for evaluation of TB-IPC practice in Tegede district, Northwest Ethiopia, 2023.

A Stakeholders	Role in the program	Interest in the evaluation	Role in the evaluation	Communication strategy	Level of importance
Healthcare providers	Providing service (program implementers)	Understanding the program implementation status and improving service delivery	Providing information	Face to face & written Formal letters	High
The service beneficiaries (clients).	Information providing about TB infection prevention and control services	Complete and quality information provision	Information providing	Face-to-face	High
Tegede district health center directors (4 HC directors)	•conducting supervision •budget allocation	• Identification of gaps & fulfilling resources	support	support	• high
Tegede District Health Office	• Met national targets • Capacity building	• Performance improvement	and	and	•medium
NGO (USAID TB Elimination)	• Technical support • Monitoring (plan, report, supervision, and review meeting)	• Service Quality improvement • Service access to Community	support	support	•medium

**Fig 1:**
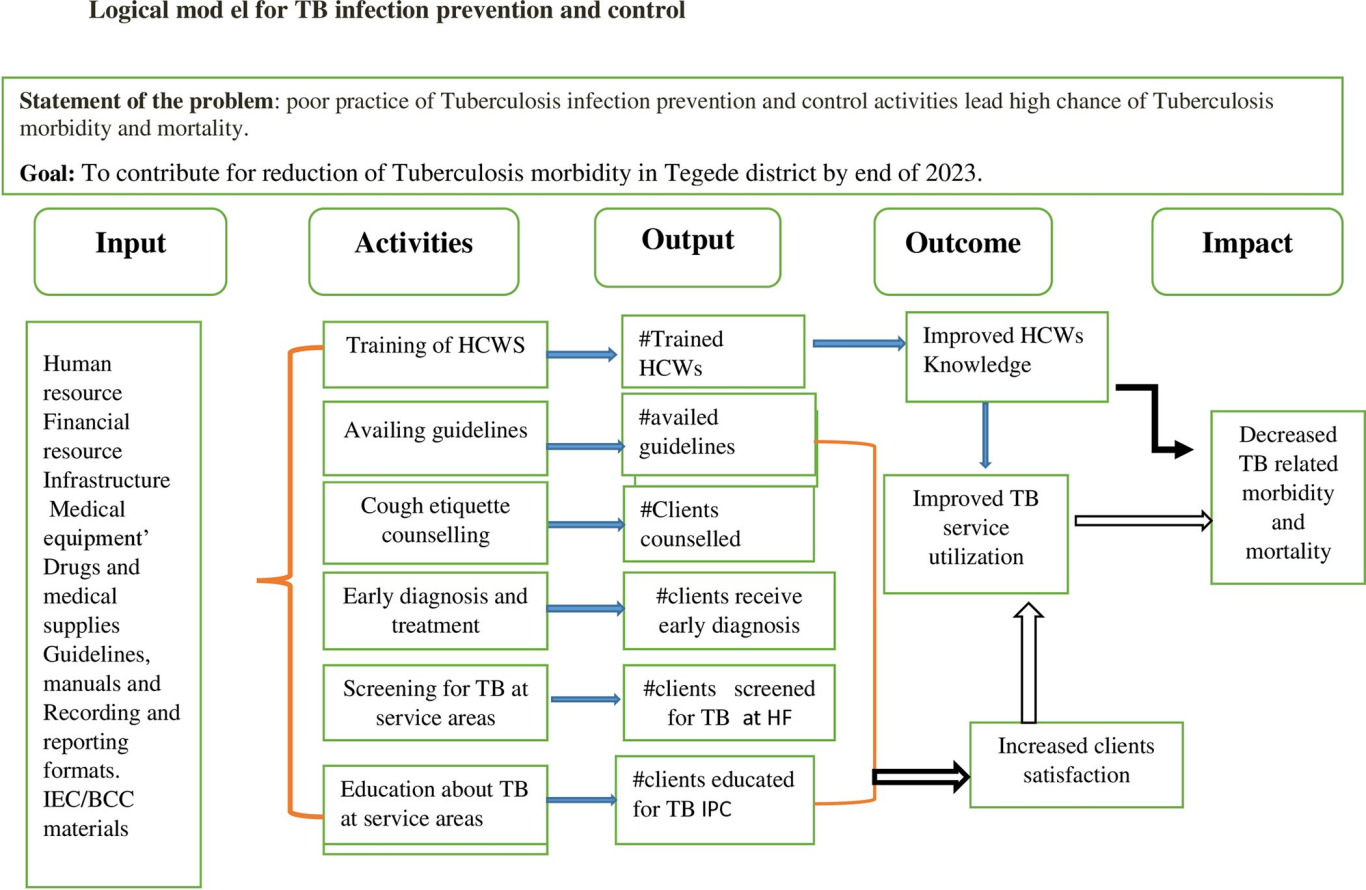
TB-IPC Logical model of Tegede district, Northwest Ethiopia, 2023.

The TB-IPC logic model is presented in Fig 1: TB-IPC Logical Model of Public Health Facilities in Tegede District, Northwest Ethiopia, 2023.

**Fig 2:**
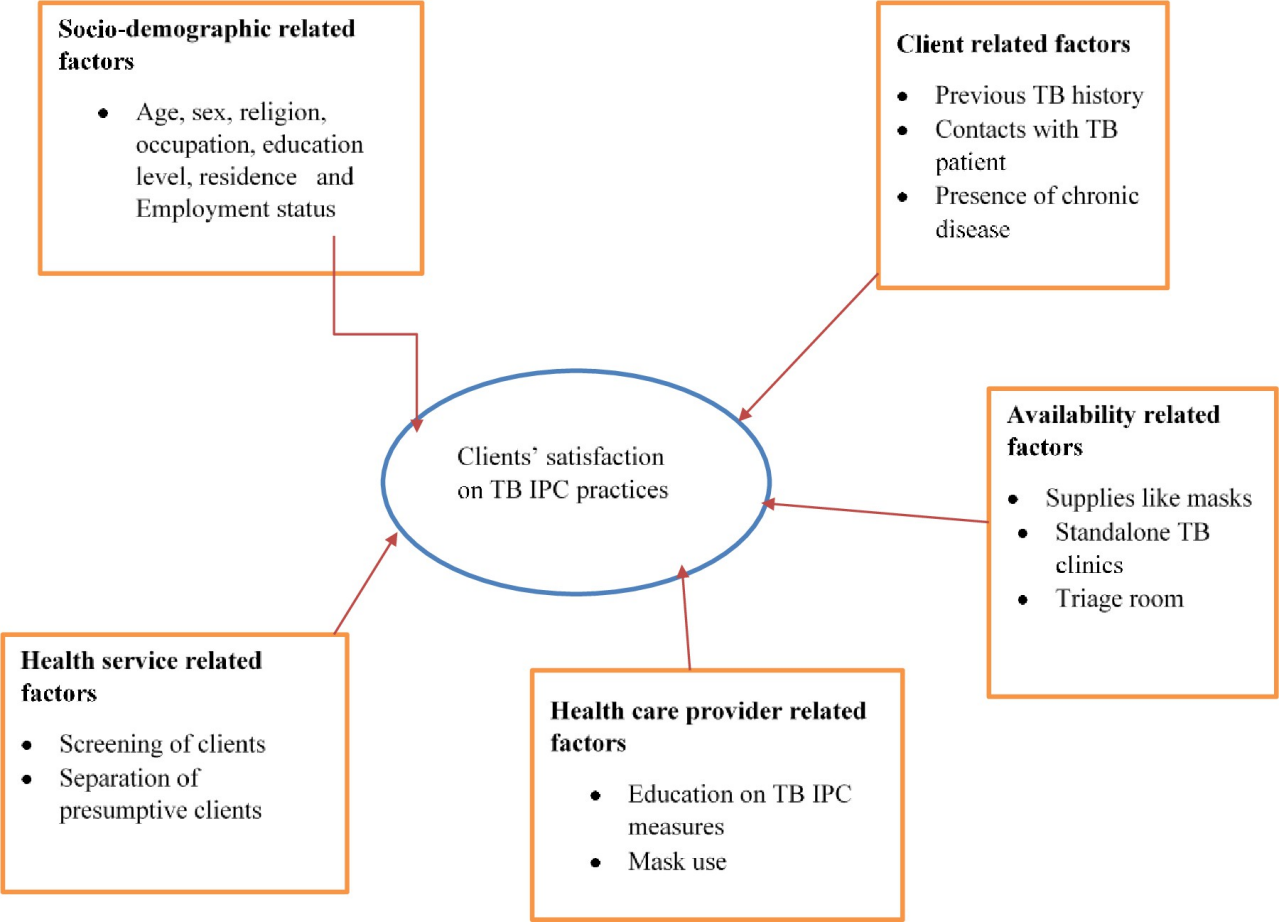
A conceptual framework to evaluate TB-IPC at public health facilities in Tegede district, northwest Ethiopia, 2023.

A conceptual framework to evaluate TB-IPC at public health facilities in Tegede district is displayed in Fig 2: A conceptual framework to evaluate TB-IPC at public health facilities in Tegede district, northwest Ethiopia, 2023 [15].

### Evaluation methods and materials

#### Evaluation area and period

The evaluation study was conducted at four health centers in Tegede district, Northwest Ethiopia, from May 1 to June 30, 2023. The district is located in the central Gondar zone, 114 km and 862 km away from Gondar City and Addis Ababa (the capital city of Ethiopia), respectively. According to the 2015 E.C. population estimates, the district’s total population was 98,927.

Tegede district has 23 health posts, 4 health centers, and 1 primary hospital that provides preventive and curative health services to the community. A total of 325 healthcare providers are assigned to provide health services in the district’s health facilities. The health facilities offer TB screening, diagnostic, and treatment services.

#### Evaluation approach

A formative evaluation approach was employed to assess TB-IPC practices at public health facilities in the Tedege District. This method aimed to identify implementation challenges and opportunities for improvement, thereby enhancing TB-IPC practices and achieving program goals. Given that the TB-IPC program is currently in the implementation stage, this approach was considered suitable for the evaluation.

#### Evaluation design

A facility-based cross-sectional study design with concurrent embedded mixed methods was conducted. Most importantly, the mixed methods approach integrates statistical analyses with data obtained from interviews, observations, or surveys, which allows researchers an opportunity to provide a more refined understanding of the topic.

#### Focus of evaluation and dimensions

The evaluation focuses on the scope of implemented TB-IPC activities, and the experiences of providers and patients. Dimensions refer to the key aspects or characteristics that are assessed to determine how well the TB-IPC practice is being implemented and achieving its goals. Dimensions help to break down complex systems into measurable parts, making it easier to evaluate and understand the program.

Therefore, availability, compliance, and acceptability dimensions were used to evaluate the implementation of TB-IPC practice. These dimensions are used to address the evaluation objective and evaluation questions [3, 16, 17].

#### Sample size determination and sampling procedures

Resource Inventory: OPD, TB clinic, and laboratory classes were observed using an adapted checklist from May 1 to June 30, 2023.

Document review: documents such as the IPC meeting agenda, focal person assignment letter, contact register, unit-TB register, TB presumptive register, AFB request, TB follow-up cards, and TB screening tools were reviewed retrospectively from January 1, 2023, to March 30, 2023.

Direct observation: a total of 60 non-participatory observations were conducted using a structured observation checklist. Five observation sessions in each of the three OPD units from four health centers were conducted to assess TB screening activities in the OPD using the opportunistic sampling technique.

### Exit interview for acceptability dimension

The required sample size was calculated by a single population proportion formula. By taking 83.8% from the same study in southern Ethiopia [17].


$$sample\;size(n)=(z_{2}^{a})2*\frac{pq}{d^{2}}N=209$$

Zα/2 = standard value of 1.96 at 95% confidence interval

d = margin of error = 5%, q = 1-p.

However, by considering the 10% non-response rate and the total sample size of 230

### The sample size for factors affecting caregiver satisfaction

The sample size for the factors associated with client satisfaction was calculated using Epi InfoTM 7 StatCal by taking the assumption of power as 80%, the confidence interval as 95%, and adding a 10% non-response rate from a study [18] (Table 2).

**Table 2:** Sample size determination for factors by using factors associated with clients’ satisfaction with TB-IPC, 2023.

Variables	Percent of outcome in unexposed	Unexposed to an exposed ratio	OR	Assumptions		Non-response rate	Sample size
				CI	Power		
Education	68.2	1:1	1.9	95	80	10%	486
Gain health education from health facilities.	74.8	1:1	2.2	95	80	10%	411
Occupational status	95.1	1:1	11.5	95	80	10%	546

The final sample size for this study was 546, based on the largest estimate obtained from two calculations: a single population proportion formula and Epi Info™ 7 StatCal. The larger of the two, 546, was used.

Key informant interview: Key informant interviews were conducted with a TB focal person, an OPD focal person, and the health center director from each of the four locations. Each individual was purposively selected for the interview. Those interviewed were chosen because of their extensive experience and training in tuberculosis infection prevention and control procedures. In total, 12 key informant interviews were conducted to gain insight from the evaluation questions.

### Data collection tools and sampling techniques

*Resource inventory*. To assess the availability of essential equipment and supplies, a checklist was developed based on established guidelines for TB-IPC. This checklist included a list of required items necessary for effective TB-IPC practices. Each facility was then evaluated during the inventory to determine the availability of these required resources. This comprehensive assessment ensured that all facility resource inventories were thoroughly reviewed.

*Document review*. Program documents, including the IPC meeting agenda, focal person assignment letters, contact register, unit TB register, TB suspect register, AFB request forms, TB follow-up cards, and TB screening tools, were reviewed retrospectively. A data extraction checklist was used to collect information on how the health centers adhered to the established guidelines.

*Sampling techniques for observation*. In this evaluation, the number of sessions observed was set using a recommendation from the Agency for Healthcare Research and Quality (AHRQ). Moreover, the sample size of health professionals observed ranged from 30% to 50% of the total number of health professionals. There were three OPDs and two healthcare providers at each health center. According to the standardized USAID’s Evaluation Toolkit, 3–5 observations were selected randomly from two healthcare providers working in the facility. A total of 60 observations were selected from 120 healthcare providers. The opportunistic sampling technique was used to select observation participants. Participants were involved in observation sessions based on the availability and willingness of individuals to participate in the study [15, 18].

*Sampling technique for exit interviews with clients*. All four health centers (4 HCs) in the Tegede district were included in the study. The sample size of 546 was allocated proportionally to each health facility based on its client flow from the same month in the previous year. Of the allocated samples in each facility, the first study participant was selected randomly using the lottery method, and to get study participants, systematic sampling techniques were used. Finally, a study participant was interviewed at every third interval using a structured interviewer administered questionnaire. The total number of clients served at health facilities in the district was 6606 in the previous year, from May 1 to June 30, 2022 [8, 19].

*Sampling technique for Key informant interview*. Purposeful sampling was conducted for key informants’ TB focal of each facility; woreda TB officers and directors of each facility to explore the views to know general information about TB-IPC practices by the key informant interview guide [3, 20].

### Data quality assurance

Five clinical nurses and two BSc nurse supervisors were recruited for interviewer-assisted data collection. All data collectors and supervisors received one-day training on data collection instruments, evaluation objectives, study participant ethics, and data collection procedures before data collection on the same day.

For direct observation, two trained male BSc public health professionals who had three and four years of work experience and were trained on TB were recruited from the nearby woreda.

Key informant interviews, resource inventory, and document review data were collected by the principal investigator.

### Data management and analysis

The questionnaires were checked daily for consistency and completeness by the principal evaluator and supervisor after data collection. Any identified issues were discussed and resolved immediately to ensure data integrity.

For handling missing or incomplete data, follow-up attempts were made to clarify or complete responses whenever feasible. If data were missing for critical variables, those specific cases were excluded from the analysis to maintain the reliability of the results. All instances of missing or incomplete data were documented along with the rationale for the chosen handling method to ensure transparency.

The data were then coded and entered into EpiData version 4.6 before being exported to SPSS version 25 for further processing and analysis. Additional checks for missing data were conducted in SPSS to confirm the completeness of the dataset prior to analysis.

Quantitative data were analyzed by exporting the coded data from EpiData Version 4.6 to SPSS Version 25 for further analysis. The results were primarily presented using frequency tables and graphs.

Variables that showed an association with the dependent variable with a p-value < 0.25 in bivariate logistic regression were considered candidates for multiple logistic regression. This higher p-value threshold was chosen to reduce the risk of Type II errors, allowing potentially relevant variables to be included that might be overlooked if a more conservative threshold (such as p < 0.05) were applied.

In the multivariate analysis, a p-value < 0.05 and a 95% confidence interval were used to declare statistical associations. Additionally, the Hosmer-Lemeshow test was conducted to assess the goodness of fit of the logistic regression model.

Qualitative data were gathered, transcribed into the text format of the Amharic language and translated into the English language. It was analyzed component by component thematically, and the result was presented in narrative form to explain the quantitative results.

### Operational definition

**Availability** refers to the physical access or presence of resources or infrastructure that meet a minimum standard. These specifications are in terms of the elements of the implementation of the initiative, such as basic equipment, commodities, the health workforce (presence and training), guidelines, and infrastructure. The availability of infrastructures for TB-IPC and the availability of supplies and drugs that are used for TB-IPC practices at health facilities. It was measured by an inventory checklist using 10 indicators. It was also given a weight of 40 percent.

**Complianc**e is the adherence of healthcare providers to TB-IPC guidelines and is measured by observation of provider-client interaction and document review using 10 indicators. It was given a weight of 30 percent.

**Acceptability:** The client’s satisfaction with the TB-IPC practices was measured by a five-point Likert scale (1 = strongly disagree, 2 = disagree, 3 = neutral, 4 = agree, and 5 = strongly agree) using 11 items. The satisfaction level was dichotomized into satisfied and dissatisfied by using the demarcation formula: (total highest score + total lowest score ÷ 2) + total lowest. Based on this formula, scores above 45 were categorized as satisfied, and those below 45 were dissatisfied. It was measured with 11 indicators and given a weight of 30 percent.

The acceptability (satisfaction) of clients with TB-IPC practices was the dependent variable.

Socio-demographic factors (age, educational status, marital status, employment status, family size, income level, client’s residence, and availability of TB-IPC supplies) were independent variables of client acceptability of TB-IPC practice.

The overall implementation of the TB-IPC practice was measured with 31 indicators using availability, compliance, and acceptability dimensions. The overall implementation level of TB-IPC practice was judged as poor (<54.9), fair (55–69.9), good (70–84.9), and very good (85–100).

### Matrix of analysis and judgment

The evaluation matrix is a tool for planning and conducting evaluations. Evaluation dimensions were weighted based on program relevance.

The evaluation score was developed together with stakeholders during the evaluability assessment based on major things that are supposed to be available for the service.

In each evaluation dimension, detailed indicators that are used to judge the performance of TB-IPC are listed and assigned weights, and the sum of the values of the indicators gives a total value for the specific dimension of evaluation. Indicators’ weight is the weight given by stakeholders before the evaluation of each selected indicator, and indicator scores are calculated using the formula (indicator score = $\frac{observed\;number*indicator\;weight\;}{expected\;number\;}$). The commutative weight obtained from each dimension of evaluation is used to judge the program’s performance based on the evaluation parameters and criteria set earlier.

The judgment matrix was determined based on the calculated indicator score; the investigator was judged poor (<54.9), fair (55–69.9), good (70–84.9), and very good (85–100), respectively [20, 21].

The interpretation of the findings was based on the dimensions and a total of 31 indicators. Finally, conclusions were drawn about the overall process evaluation of TB-IPC practice, and each health facility received a customized report detailing the findings specific to their facility and the recommended actions for improvement.

Ethical clearance was obtained from the Institute of Public Health ethical review committee at the University of Gondar, College of Medicine and Health Sciences, with reference number IPH/2513/2023, on April 11, 2023, and supporting letters were taken from the Tegede Woreda Health Office and other relevant organizations in the study area. Written informed consent was obtained from the respondents, and the confidentiality of participants’ information was maintained by anonymous data. The participants were informed that participation in this study is voluntary, and you can withdraw at any time without any consequences.

### Evaluation dissemination plan

Once the data collection and analysis were completed, a report was prepared using a standardized structure. The final version of the evaluation results was conducted with stakeholders. A draft report was shared and discussed initially with stakeholders in Tegede district health facilities and advisors for their comments. Incorporation of all relevant comments was done, and after the approval of the thesis, hard copies of the evaluation report were disseminated to the University of Gondar, Tegede district health office, zonal health department, and other stakeholders. Finally, the paper was sent for publication in a peer-reviewed journal.

## Result

In this evaluation study, we conducted a resource inventory, reviewed documents from January 1, 2023, to March 30, 2023, observed 60 healthcare providers during client interactions, completed 525 client exit interviews with a 96.2% response rate, and interviewed 12 key informants.

### Availability

A total of 105 staff are employed in four health centers; among these, 77 (73.3%) are healthcare providers and 28 (26.7%) are administrative staff. From these, there are four general practitioners, eight health officers, fifteen midwives, thirty-three nurses, nine laboratory professionals, eight pharmacy professionals, and twenty-eight administrative staff in all four health centers.

Among 77 healthcare providers, only 5 (6.5%) were trained in TB-IPC training programs. This was supported by a key informant interview.

“*Focal people only received TB training. The training should be delivered to other professionals as well, because when we leave the facility for different purposes, the client would not get the service appropriately.”*

        ***[28-year-old male BSc nurse, TB focal person]***

Findings from resource inventory indicated that from a total of four public health centers, 2 (50%) had sputum collection caps, 4 (100%) had surgical masks, 3 (75% had N-95 masks), 4 (100%) had isoniazid, and 4 (100%) had isoniazid with rifampicin. Additionally, all health centers had AFB microscopes, and 3 (75%) of health centers had microscope reagents during the day of resource inventory based on inventory assessment results.

This finding was similar to a key informant interview.

“*Resources required for TB-IPC have not been fully availed*. *Resources that are not available now are the triage room*, *reagents for AFB microscopy*, *weighing scale*, *and adult MUAC tape*. *Due to the absence of reagents from EPSA and lack of commitment of HC heads to fulfill”*

        ***[32-year-old male, public health TB focal person]***

Five (6.5%) of healthcare providers received training about TB. Concerning TB registration and reporting formats, 3 (75%) of them had a TB suspect register and a TB follow-up card, but all health centers had contact registers, unit TB registers, an AFB request form, a TB screening tool, and a TB report format.

### Summary of TB IPC program performance process on evaluation of availability indicators

The overall proportion of resource availability to implement tuberculosis prevention and control practices at public health facilities was found to be 50.4%. It was rated as poor based on the judgment criteria. This finding was supported by the key informant interviews.

“*TB infection prevention and control practices are poorly implemented due to different reasons like lack of resources and low concern for TB-IPC practices; TB-IPC practices are poorly implemented due to work overload and health professionals’ negligence on TB-IPC practice.”*

***[30-year-old male, untrained BSc nurse professional]***

A judgment summary of the availability dimension is presented in Table 3.

**Table 3:** Judgment summary of availability dimension of TB-IPC practices at public health facilities in Tegede district, Northwest Ethiopia, May 2023.

S.No	Availability indicators	Expected in #	Observed in #	Weight (W)	Score (S)	Ach.in % $\frac{S}{W}*100$	Judgment Parameter
1	Proportion of trained health professionals for the TB-IPC training program	77	5	6	0.4	6.5	Poor
2	Proportion of health facilities with documented TB-IPC program plan.	4	1	4	4	100	Very good
3	Proportion of health facilities with the TB-IPC committee.	4	2	4	2	50	Poor
4	Proportion of health facilities with essential medications (Isoniazid and Rifampicin) available for TB-IPC during the study period.	4	4	4	4	100	Very good
5	The proportion of health facilities with medical supplies (sputum cap, surgical mask, and N-95 mask) available for TB IPC during the study period.	4	2	3	1.5	50	Poor
6	The proportion of health facilities with ventilated waiting spaces.	4	3	3	2.3	75	Good
7	The proportion of health facilities with cougher triage.	4	0	4	0	0	Poor
8	The proportion of health facilities with laboratory service for TB diagnosis (AFB).	4	2	4	2	50	Poor
9	The proportion of health facilities with a standalone TB clinic.	4	2	4	2	50	Poor
10	The proportion of health centers IPC meetings conducted based on the schedule	4	2	4	2	50	Poor
**Overall implementation of the TB-IPC practices for availability dimension**				40	20.1	50.35	Poor

### Compliance with national TB-IPC guidelines

#### Document review

The IPC meeting agenda, focal person assignment letter, contact registers, unit TB registers, TB presumed registers, AFB request forms, TB follow-up cards, and TB screening tools in four health centers were reviewed. Based on the review results, half of the health centers had an IP committee, an IPC meeting, and a TB focal person. One-fourth (1/4) of the health center had only a TB-IPC plan, conducted a TB risk assessment, and screened the facility staff for TB. The other review finding revealed that none of the staff had developed TB in the facility, and none of the health centers isolated patients with coughs early from other patients.

*“All household contacts with TB cases are not cooperative enough to come to the facility to be screened for TB, but all those eligible will not get TB prophylaxis because some of the contacts do not voluntarily take prophylaxis, and sometimes supply shortages also occur.”*

***[29-year-old male, BSc nurse, TB focal person]***

In the assessment of TB infection control practices at health facilities, 45(75%) of health workers asked about TB symptoms during client examinations, and 60 (100%) of OPD room windows and doors were open. However, there were significant gaps: no health workers asked about coughs, separated coughers in the waiting area, or had respirators available for staff in high-risk areas (Table 4).

**Table 4:** Recording and reporting of TB-IPC activities in public health centers of Tegede district, Northwest Ethiopia, May 2023.

S.No	Activities observed	Frequency	Percentage
1	Does the health worker ask for coughs for clients entering the facility?	0	0
2	Does the health worker separate coughers in the waiting area?	0	0
3	Do the health workers ask for TB symptoms during examinations of clients at OPD?	45	75.0
4	Do the health workers wear masks during examinations of clients at OPD?	15	25.0
5	Do OPD room windows and doors open?	60	100.0
6	Does the OPD room have a functional mechanical ventilation system?	45	75.0
7	Does the waiting area have a functional mechanical ventilation system?	15	25.0
8	Is there a separate/designated sputum collection area?	30	50.0
9	Are respirators available for staff working in high-risk areas?	0	0
10	Is the laboratory sputum smear preparation area cross-ventilated?	45	75.0
11	Is the laboratory room size adequate?	45	75.0
12	Are outdoor waiting areas large enough to accommodate patients without crowding?	0	0
13	Is there cross-ventilation in OPDs, including TB clinics?	30	50.0
14	Is there cross-ventilation in the waiting area(s)?	60	100
15	Is there mechanical ventilation in OPDs, including TB clinics?	45	75.0

### Observation of activities

Observations were conducted to assess TB-IPC practices among healthcare providers in the facility. All healthcare providers opened the windows and doors of their rooms, ensuring cross-ventilation in the waiting areas during the observation period. Additionally, 45 (75%) of healthcare providers asked about TB symptoms during client examinations at the OPD. The OPD rooms were equipped with functional mechanical ventilation, and the sputum smear preparation laboratory areas were also cross-ventilated and adequately sized.

However, significant gaps were identified: no healthcare providers asked clients about coughing upon entry to the facility, and there was no separation of coughing clients in the waiting area. Furthermore, respirators were not available in high-risk areas, and outdoor waiting areas were insufficiently sized to accommodate patients without overcrowding (Table 5).

**Table 5:** Observation on compliance of public health facilities in Tedege district, Northwest Ethiopia, May 2023.

S.No	Activities observed	Frequency	Percentage
1	Does the health worker ask for coughs for clients entering the facility?	0	0
2	Does the health worker separate coughers in the waiting area?	0	0
3	Do the health workers ask for TB symptoms during the examination of clients at OPD?	45	75.0
4	Do the health workers wear masks during examinations of clients at OPD?	15	25.0
5	Do OPD room windows and doors open?	60	100.0
6	Does the OPD room have a functional mechanical ventilator?	45	75.0
7	Does the waiting area have a functional mechanical ventilator?	15	25.0
8	Is there a separate/designated sputum collection area?	30	50.0
9	Are respirators available for staff working in high-risk areas?	0	0
10	Is the laboratory sputum smear preparation area cross-ventilated?	45	75.0
11	Is the laboratory room size adequate?	45	75.0
12	Are outdoor waiting areas large enough to accommodate patients without crowding?	0	0
13	Is there cross-ventilation in OPDs, including TB clinics?	30	50.0
14	Is there cross-ventilation in the waiting area(s)?	60	100
15	Is there mechanical ventilation in OPDs, including TB clinics?	45	75.0

### Summary of TB-IPC practices performance process on evaluation of compliance indicators

The compliance dimension was measured using 10 indicators and had a weight value of 30%, scored 20.2%, and achieved 67.2%. It is set as fair according to the judgment parameter (see Table 6).

**Table 6:** Judgment summary of compliance dimension of TB-IPC practices in Tegede district public health facilities, Northwest Ethiopia, May 2023.

S.No	Compliance indicators	Expected in #	Observed in #	Weight (W)	Score (S)	Ach.in % $\frac{S}{w}*100$	Judgment Parameter
1	The proportion of healthcare providers screened for TB in the last quarter.	4	1	2	0.5	25.0	Poor
2	The proportion of health facilities that have done facility TB IPC risk assessment using a facility TB risk assessment checklist.	4	1	2	0.5	25.0	Poor
3	The proportion of health facilities posted by IEC regarding cough etiquette in the waiting area, OPD, and lab units.	4	3	3	2.3	75.0	Good
4	The proportion of health facilities that had cross-ventilated OPDs	4	3	4	3	75.0	Good
5	The proportion of health facilities that had mechanical ventilation at OPD, TB clinic, lab unit, and waiting area.	4	3	2	1.5	75.0	Good
6	A proportion of healthcare providers used personal protective equipment at the time of data collection.	60	15	4	1	25.0	Poor
7	Proportion of HH contacts of bacteriologically confirmed TB patients that were investigated for TB	41	38	4	3.7	92.8	Very good
8	Proportion of HH contacts eligible for TPT who were prescribed treatment	8	7	3	2.6	87.7	Very good
9	Proportion of HH contacts eligible for TPT, who initiated TPT	7	6	3	2.6	85.7	Very good
10	Proportion of HH contacts who initiated TPT and completed TPT	6	5	3	2.5	83.3	Good
**Overall implementation of the TB-IPC practices for compliance dimension**				30	20.2	67.2	Fair

### Acceptability

#### Socio-demographic characteristics of respondents

Among the total respondents, 155 (29.5%) were from Soroka Health Center, and 279 (53.1%) were males. The age of respondents ranged between 18 and 68, and the mean age was 28.4. All of the respondents were Amharic language speakers. Most identified as Orthodox Christian followers 497 (94.7%) were orthodox Christian religion followers, and 229 (43.6%) could read and write.

Regarding marital status, more than half (58.7%) of respondents married. Additionally, 334 respondents (63.6%) earned an income between 2501 and 3500 Ethiopian birr (Table 7).

**Table 7:** Socio-demographic characteristics of respondents at public health centers in Tegede district, Northwest Ethiopia, May 2023.

Variable	Categories	Frequency (N = 525)	Percentage (%)
**Facility**	Kirakira HC	111	21.1
	Ergoye HC	126	24.0
	Soroka HC	155	29.5
	Adet HC	133	25.3
**Sex**	Male	279	53.1
	Female	246	46.9
**Age**	Less than 35	442	84.2
	Greater than 35	83	15.8
**Language**	Amharic	525	100.0
**Religion**	Orthodox	497	94.7
	Muslim	28	5.3
**Educational status**	Illiterate	112	21.3
	Read and write	229	43.6
	Primary education	132	25.1
	Secondary education	30	5.7
	College and above	22	4.2
**Marital status**	Single	185	35.2
	Married	308	58.7
	Widowed	2	.4
	Divorce/separate	30	5.7
**Occupation**	Government employee	30	5.7
	Farmer	202	38.5
	Merchant	35	6.7
	House wife	126	24.0
	Daily laborer	50	9.5
	Unemployed	82	15.6
**Family monthly income**	501–1000	11	2.1
	1001–2500	84	16.0
	2501–3500	334	63.6
	>3500	96	18.3
**Residence**	Urban	90	17.1
	Rural	435	82.9

**Fig 3:**
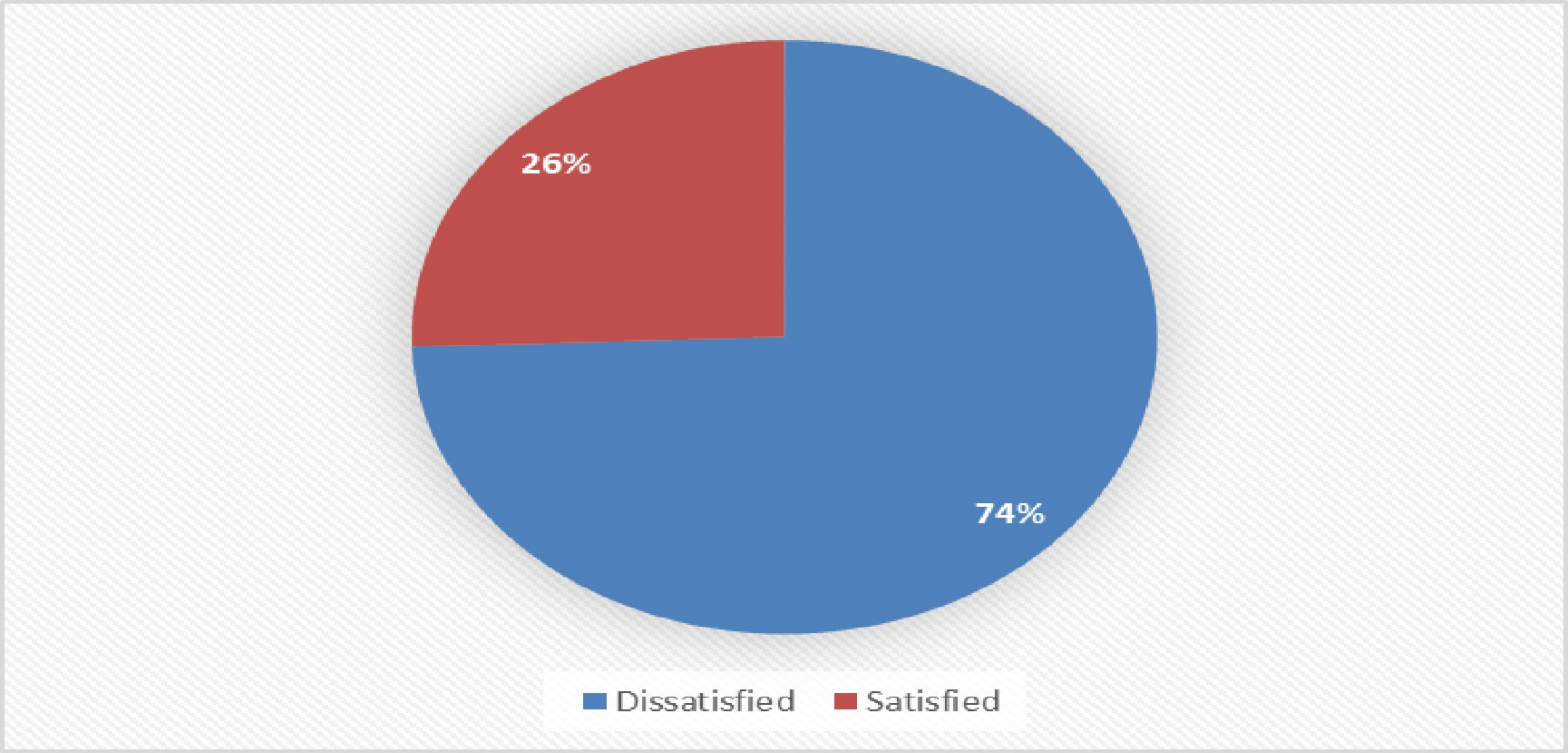
Clients overall satisfaction of clients with TB-IPC practices at public health centers of Tegede district, May 2023.

Clients overall satisfaction with TB-IPC practices at public health centers in Tegede district is presented in Fig 3: Clients overall satisfaction with TB-IPC practice at public health centers in Tegede district, May 2023.

Fig 3 shows that 134 (25.5%) clients (95% CI = 21.96%, 29.4%) were satisfied with TB-IPC practices at Tegede district public health centers.

### Factors affecting client satisfaction with the TB-IPC practices in public health facilities of Tegede District

Religion, education, occupation, residence, and family monthly income were significant in binary logistic regression with a 95% confidence interval, and they were candidate variables for multivariable analysis.

In multivariable logistic regression analysis, occupation was statistically significant in predicting TB-IPC acceptability (p < 0.05).

Farmers had an odds ratio (AOR) of 22.5 for TB-IPC acceptability compared to government-employed clients (AOR = 22.498, 95% CI: 2.424–208.776), indicating that farmers were significantly more likely to accept TB-IPC practices. In contrast, merchants had lower odds of TB-IPC acceptability (AOR = 0.487, 95% CI: 0.243–0.977), suggesting they were less likely to accept these practices compared to government employees.

Housewives showed a strong likelihood of accepting TB-IPC practices with an AOR of 15.3 (95% CI: 4.421–51.939), indicating they were significantly more inclined to accept these measures compared to their counterparts. Conversely, unemployed clients had much lower odds of TB-IPC acceptability (AOR = 0.144, 95% CI: 0.031–0.655) when compared to government-employed clients, suggesting a significant barrier to acceptance among this group.

These results indicate that occupation plays a crucial role in determining TB-IPC acceptability, with notable variations across different occupational groups (presented in Table 8).

**Table 8:** Binary and multivariable logistic regression analysis result of clients’ satisfaction with the acceptability of TB program service delivery in Tegede District public health facilities, Northwest Ethiopia, 2023.

Variables	Satisfaction				p-value
	Dis Satisfied	Satisfied	COR(95%CI)	AOR(95%CI)	
	Frequency (%)	Frequency (%)			
Occupation					
Government employee	5(1.23)	25(18.66)	1	1	0.000
Farmer	180(46.04)	22(16.42)	13.636(4.643,40.047)	22.498(2.424,208.776)	0.006**
Merchant	4(1.02)	31(23.13)	0.333(0.172,0.644)	0.487(0.243,0.977)	0.043**
House wife	94(24.04)	32(23.88)	21.136(6.691,66.765)	15.273(4.421,51.939)	0.000**
Daily laborer	48(12.28)	2(1.49)	0.93(0.493,1.747)	1.251(0.634,2.472)	0.518
Unemployed	60(15.35)	22(16.42%)	0.114(0.025,0.507)	0.144(0.031,0.655)	0.012**
Educational status					
Illiterate	108(27.62)	4(2.99)	1	1	0.000
Can read and write	189(48.34)	4029.85)	0.011(0.003,0.045)	0.354(0.030,4.109)	0.406
Primary education	82(20.97)	50(37.31)	0.062(0.022,0.179)	1.675(0.176,15.905)	0.653
Secondary education	7(1.79)	23(17.16)	0.179(0.062,0.516)	3.676(0.389,34.765)	0.256
College and above	5(1.28)	17(12.69)	0.966(0.261,3.573)	5.455(0.600,49.592)	0.132

COR; crude odds ratio; AOR; adjusted odds ratio; * * Variables associated with the satisfaction of clients of the TB IPC program in multi-logistic regression analysis.

The overall acceptability of the TB-IPC practices was rated poor, with a score of 43.4% (Table 9).

**Table 9:** Judgment summary of the acceptability dimension of the TB-IPC practices in Tegede district public health facilities, Northwest Ethiopia, May 2023.

S.No	Acceptability indicators	Expected in #	Observed in #	Weight (W)	Score (S)	Ach.in % $\frac{S}{W}*100$	Judgment Parameter
1	The proportion of clients who are satisfied with screening for TB in the examination room.	525	347	3	2.0	66	Fair
2	The proportion of clients satisfied with the separation of cougars in the facility.	525	123	2.5	0.6	23.6	Poor
3	The proportion of clients satisfied with the cleanliness of the facility.	525	198	2.5	0.9	37.6	Poor
4	The proportion of clients satisfied on the ventilation of OPD	525	121	2.5	0.6	23.2	Poor
5	Proportion of clients satisfied with openness of doors and windows during examination.	525	363	3	2.1	69	Fair
6	The proportion of clients satisfied with the waiting area has enough space and adequate ventilation for the clients.	525	322	3	1.8	61.3	Fair
7	Proportion of clients satisfied with the existence of a separate and a standalone TB clinic place.	525	145	3	0.8	27.7	Poor
8	Proportion of clients satisfied with waiting time for medical care	525	370	3	2.2	71.7	Good
9	The proportion of clients satisfied with education on cough etiquette during coughing and sneezing in the facility.	525	292	2.5	1.4	55.6	Fair
10	Proportion of clients satisfied with the improvement in condition after initiation of treatment.	525	78	2	0.30	15.0	Poor
11	Proportion of clients satisfied with screening for TB in the examination room.	525	60	3	0.3	11.3	Poor
**Overall implementation of TB- IPC practices for acceptability dimension**				30%	13.0	43.4	Poor

The implementation process of TB-IPC practices achieved a score of 54.4 out of 100, which is 54.4% of the total weight. Based on the preset judgment parameters, this overall performance was deemed “needs urgent improvement" (Table 10).

**Table 10:** Summary of overall TB-IPC practice at public health facilities in Tegede district, Northwest Ethiopia, May 2023.

S.No	Dimensions	Relative weight (W)	Score(S)	Achv.in % (S/W*100)	Judgment parameter
1	Availability	40	20.1	50.4	Poor
2	Compliance	30	21.2	67.2	Fair
3	Acceptability	30	13.0	43.4	Poor
The implementation process of TB-IPC practices		100	54.4	54.4	Poor = Needs urgent improvement

## Discussion

The results of this evaluation revealed that the overall process of TB practice implementation in public health clinics in the Tegede district was poor (54.4%). Moreover, the availability of resources was poor (50.4%). According to the judgment criteria, healthcare providers’ compliance with national TB guideline recommendations and client acceptability were judged as fair (67.2%) and poor (43.4%), respectively. The implementation of TB-IPC practices requires immediate remediation.

### Availability dimension

The study found that only 50.4% of the necessary resources for TB-IPC were available. This is consistent with findings from other regions in Ethiopia, where resource availability for TB-IPC was similarly low. For instance, a study conducted in Southern Ethiopia reported an implementation rate of only 34% for TB-IPC practices [22]. This poor availability is often attributed to inadequate infrastructure and lack of financial resources, a challenge not unique to Ethiopia but prevalent in many low-income countries [23].

### Compliance with the TB national guideline

The compliance rate of 67.2% suggests that while some guidelines are being followed, significant non-compliance exists. This aligns with research in other Ethiopian healthcare settings, where compliance with TB-IPC guidelines was found to be inconsistent [24]. Internationally, studies have also shown varying compliance rates, often linked to staff training and resource availability [25]. The lack of trained personnel specifically impacts compliance, as only 6.5% of healthcare providers in this study had received TB-IPC training.

### Acceptability of TB infection prevention and control

The study reported that only 25.5% of clients (95% CI = 22.0%, 29.44%) were satisfied with TB-IPC practices. This low satisfaction rate echoes findings from other studies indicating that patient perceptions of care quality are often linked to the availability of resources and adherence to safety protocols [26]. In a similar context, a study in Kenya noted that patient satisfaction improved significantly when health facilities adhered to IPC protocols [27].

According to the findings of this study, occupation was a statistically significant variable influencing clients’ satisfaction with TB-IPC practices in public health centers in the Tegede district (p < 0.05). The varying levels of TB-IPC acceptability among different occupations underscore the importance of tailoring health interventions to specific groups. Farmers and housewives, who typically have closer ties to community health initiatives and a better understanding of health risks, are more receptive to TB-IPC practices. In contrast, merchants and unemployed individuals may benefit from targeted outreach and education to enhance their awareness and acceptance of TB prevention measures.

Understanding these dynamics can inform public health strategies, ensuring that interventions are appropriate for the unique challenges and perspectives of each occupational group. By addressing the barriers faced by merchants and unemployed individuals, health programs can improve overall TB-IPC acceptability and effectiveness.

Additionally, socio-demographic factors, such as education and employment status, are known to influence health-seeking behavior and satisfaction levels. Previous studies have shown that individuals with higher educational attainment are more likely to understand and effectively utilize health services [28, 29].

### Limitations of the evaluation

The satisfaction of clients might be overstated because the clients who are dissatisfied with the service may not come to the health center.

The other limitation of this evaluation was the Hawthorne effect during observation of healthcare.

Providers practice infection prevention to minimize this bias. Three client gaps were observed in between observations.

## Conclusion

These findings underscore the urgent need for strengthening TB-IPC practices within public health facilities in Tegede district. The suboptimal implementation rates highlight systemic issues, including inadequate training, insufficient resources, and low prioritization of TB-IPC by healthcare providers. Better to enhance training programs for healthcare workers to improve compliance with TB-IPC guidelines. Investment in human resources, including training for all health professionals rather than just focal persons, and better to allocate resources, including the provision of essential equipment and medications could significantly improve practice outcomes. This study adds valuable data to the body of literature on TB-IPC in Ethiopia and highlights specific areas for improvement.

## Supporting information

S1 File (RAR)

## Abbreviations

AFB: Acid-Fast Bacilli
BCC: Behavioral Change Communication
DR: : Drug Resistance
EPSA: Ethiopian Pharmaceutical Supply Agency
HC/HF: Health Centers/Health Facilities
HH: Household
HIV: Human Immunodeficiency Virus
ICE: Information Communication and Education
IPC: Infection Prevention and Control
MDR: Multidrug Resistant
MUAC: Mid Upper Arm Circumference
NGO: Non-Governmental organization
OPD: Outpatient Department
Pre-XDR: Pre-Extensively Drug Resistance
RH: Rifampicin and Isoniazid
TB: Tuberculosis
TPT: Therapeutic Preventive Therapy
WHO: World Health Organization
XDR: Extensively Drug Resistance
